# Transport from the wild rapidly alters the diversity and composition of skin microbial communities and antifungal taxa in spring peeper frogs

**DOI:** 10.3389/frmbi.2024.1368538

**Published:** 2024-04-19

**Authors:** Lauren P. Kane, William G. Van Bonn, Francis J. Oliaro, Christian F. Edwardson, Malissa Smith, Lee J. Pinnell

**Affiliations:** ^1^Animal Care and Science Division, John G. Shedd Aquarium, Chicago, IL, United States; ^2^Department of Animal Health, Pittsburgh Zoo & Aquarium, Pittsburgh, PA, United States; ^3^Department of Aquatic Sustainability, Georgia Aquarium, Atlanta, GA, United States; ^4^Veterinary Education, Research, and Outreach Program, Texas A&M University, Canyon, TX, United States

**Keywords:** amphibian, microbiome, skin, antifungal taxa, managed environment

## Abstract

Amphibians are routinely collected from the wild and added into managed care and public display facilities; however, there is a gap in understanding how these practices might alter the diversity and composition of skin microbial communities on these animals. The aim of this study was to evaluate and compare skin microbial communities of spring peeper frogs (*Pseudacris crucifer*) from acquisition in the wild through the end of their quarantine period and identify microbial taxa with antifungal properties. From an original group of seventy-six frogs, cohorts of ten were swabbed when acquired in the wild, upon transport from the wild, and swabbed throughout a 9-week quarantine period while under managed care. An immediate loss of microbial richness and diversity was evident upon transfer of the frogs from their original environment and continued throughout subsequent sampling time-points during quarantine. Importantly, antifungal taxa comprised significantly more of the overall skin community after the frogs were moved from the wild, largely due to members of the family Moraxellaceae. Overall, our findings demonstrate that amphibian skin microbiome changes immediately on removal from the wild, and that these changes persist throughout quarantine while being housed under managed care. This may play a pivotal role in the development of dermatological disease and have implications in the health and immune function of amphibians.

## Introduction

Amphibians are routinely collected from the wild and added into managed care and public display facilities. While these managed collections serve critical roles in education, research, and conservation efforts, there remains a knowledge gap in understanding how the move from a wild environment to a managed one alters the skin of amphibians and their associated microbial communities. Amphibian skin serves as a thin, permeable organ that serves a variety of critical physiological functions like respiration, ion transport, osmoregulation, while also playing an important role in pathogen defense (Lillywhite, 2006; Campbell et al., 2012; Varga et al., 2019). However, due to its morphological features it is also more susceptible to environmental variations like temperature fluctuations, moisture levels, and habitat alterations (Kueneman et al., 2014; Ethier et al., 2021). Amphibian skin houses various glands that exhibit specificity both in terms of species and life stage (Knutie et al., 2017; Pasmans and Martel, 2019). Among the noteworthy glands are the holocrine serous glands, responsible for secreting a diverse array of bioactive molecules, including antimicrobial peptides. These molecules play a crucial role in regulating the microbial population residing on amphibian skin (Pasmans and Martel, 2019; Jiménez et al., 2022).

The microbial communities on amphibian skin play a crucial role in bolstering host immunity and fortifying defenses against potential pathogens. These communities create a dynamic and symbiotic relationship with their amphibian host through the generation of antimicrobial and antifungal molecules and the stimulation of host immune responses (Mangoni et al., 2001; Kueneman et al., 2014; Woodhams et al., 2016). Damage to the skin disrupts the critical functions of microbial communities and predisposes the affected individual to loss of homeostasis and potential death. Understanding the interplay between amphibian skin, its unique glands, and the associated microbial communities is pivotal in comprehending the broader implications for amphibian health and well-being.

Skin microbial communities exhibit remarkable diversity within amphibians, underscoring the critical need for a comprehensive understanding of their ecology to unravel the importance of the microbial interactions occurring on amphibian skin. While bacterial populations in reptiles and amphibians are often broadly categorized as gram-positive or gram-negative (Wellehan and Divers, 2019), a more nuanced exploration is imperative. In general, commensal bacteria such as members of the genera *Aeromonas, Pseudomonas, Proteus, and Escherichia* are routinely cultured from the skin of healthy amphibians (Whitaker and Wright, 2019). However, recognizing that the presence of two significant pathogens, iridoviruses and the fungus *Batrachochyutrium dendrobatidis (Bd)*, have recently been associated with changes in the diversity and composition of the skin microbiome (Chen et al., 2022; Sun et al., 2023) underscores the need for a holistic understanding of microbial interactions. Further, amphibian skin microbiome produced metabolites have been shown to inhibit *Bd* zoospore development (Loudon et al., 2014; Walke and Belden, 2016) and the presence of certain anti-fungal microbial taxa on uninfected frogs has been shown to reduce morbidity after exposure to *Bd* (Harris et al., 2009; Walke and Belden, 2016). A comprehensive understanding of the ecology of these microbial communities is crucial for advancing our knowledge of amphibian health and developing effective strategies for the conservation and management of amphibian populations.

In the present study, we used 16S rRNA sequencing to investigate the diversity and composition of skin microbial communities on spring peeper frogs (*Pseudacris crucifer*) in response to transport from the wild and management under human care. Sampling occurred in the wild and over the course of a typical quarantine period applied to amphibians being introduced to managed environments at a large public display aquarium. Importantly, we also investigated the impact of collection and handling on the diversity and predominance of microbial taxa with recognized antifungal activity within the overall community.

## Materials and methods

### Study design and sample collection

Spring peeper frogs (*Pseudacris crucifer*) were visually identified at night based on their natural habitat behaviors and collected from the Shawnee National Forest in Harod, Illinois (n = 76). All frogs were caught by gloved hands, and a random subsample (n = 10) were swabbed with sterile cotton tipped applicators (Puritan Medical Company LLC, Guiliford, ME). Frogs were immediately transferred to sterile 50 milliliter sterile conical-bottom tubes (Cole-Parmer, Vernon Hills, IL). A second random subsample of frogs (n = 10) was then collected again by swabbing frogs with a different cotton tipped applicator as they were being moved from their individual, sterile 50mL tubes to a second individual container. These individual containers were disinfected with bleach before collection, and were perforated with a bare bottom. Once swabbed a second time, frogs were held in their individual containers in a cooler until being released into their quarantine enclosure at Shedd Aquarium.

In quarantine, frogs were housed in 10-gallon glass tanks on a wetted paper towel as substrate. Habitats also contained polyvinyl chloride tubes and fake plants as hides. All the frog habitats were spot cleaned weekly during the 30-day quarantine period. Frogs were fed pinhead crickets, small crickets, and fruit flies dusted with calcium supplementation without vitamin D3 (ReptiCalcium, ZooMed Laboratories, Inc., San Luis Obispo, CA). During quarantine, a random subsample of frogs (n = 10) were swabbed during weeks 1, 2, 4, 7, and 9 of a 9-week quarantine period. During the second week of quarantine, the frogs were given a single topical treatment of ivermectin (2mg/kg; Noromectin, Norbrook Inc. USA, Lenexa, KS) topically along the dorsum, as a standard quarantine antiparasitic. Sampling during the quarantine period occurred by coaxing the frog to jump into a sterile conical tube for prevention of direct handling. The ventral and dorsal skin of each frog was sampled with the same cotton swab, placed into sterile cryovials, and stored at -80C until DNA extraction. Once the swabs were collected, the tubes were opened in their individual enclosures and the frogs jumped out, without requiring handling. Frogs were not individually identified and a random subsample of ten were sampled at each timepoint during the study, and whether the same individual was swabbed at multiple timepoints was unknown. As such, microbial communities at each timepoint were not considered to be repeated measures of dependent communities.

The frogs in this study were collected under permit from the Illinois Department of Natural Resources (Permit Number: A15.1006).

### DNA isolation, 16S rRNA library preparation and sequencing

DNA was isolated from skin swabs using the MoBio PowerSoil HTP kit (MO BIO), according to manufacturer instructions. Bacterial and archaeal DNA was amplified using primer constructs (515f/806rB) targeting the V4 region of the 16S rRNA gene (Walters et al., 2016). The constructs contain Illumina-specific adapters followed by 12 bp Golay barcodes on each forward primer, primer pads, and linkers as well as the template-specific PCR primer at the 3′ end. PCR was performed in replicate 25 µl reactions containing 12.5 µl 5PRIME Hot-Start 2× MasterMix (QuantaBio), 0.2 µM final concentrations of forward primer 515f and reverse primer 806rB, 2 µl of template DNA and nuclease-free water to equal 25 µl. Thermal cycling conditions were carried out as follows: 94°C for 3 minutes, 35 cycles at 94°C for 45 seconds, 50°C for 60 seconds and 72°C for 90 seconds, with a final extension of 10 min at 72°C. After PCR, replicate amplicons were combined and 5 µl of each were electrophoresed in 1.8% agarose gels to confirm amplification of the V4 region. Amplicon concentrations were quantified using PicoGreen (Life Technologies) and a microplate reader (Tecan) then pooled equally via automated liquid handling (ePMotion, Eppendorf). The pooled amplicon library was purified with UltraClean PCR Clean-Up Kit (MO BIO Laboratories) then quantified using a Qubit™ 3.0 fluorometer and Qubit™ dsDNA HS Assay Kit (Life Technologies). The molarity of the pooled library was calculated and diluted to 2 nM before denaturation and further dilution to a loading concentration of 8 pM. Paired-end sequencing for a total of 500 cycles was conducted on the Illumina MiSeq platform using custom sequencing primers described previously (Caporaso et al., 2012) with the addition of a 10% PhiX Control library (Illumina) to increase sequence diversity.

### Bioinformatics

Demultiplexed 16S rRNA gene sequence reads were imported into QIIME2 version 2022.2 (Bolyen et al., 2019). Amplicon sequence variants (ASVs) were generated using DADA2 (Callahan et al., 2016), which also filtered reads for quality, removed chimeric sequences, and merged overlapping paired-end reads. Forward reads were trimmed at 19bp, and reverse reads were trimmed at 20bp, while forward reads were truncated at 250bp and reverse reads were truncated at 248bp. Taxonomy was assigned using a Naïve Bayes classifier trained on the SILVA 138 SSU NR 99 database (Quast et al., 2013), where sequences had been trimmed to only include the V4 region bound by the 515f/806r primer pair. Reads mapping to chloroplast and mitochondrial sequences were removed from the ASV table and representative sequences, and a mid-point rooted phylogenetic tree was generated using ‘qiime alignment mafft’, ‘qiime alignment mask’, and ‘qiime phylogeny fasttree’ under default settings. The ASV table, representative sequences, and mid-point rooted tree were then imported into phyloseq (McMurdie and Holmes, 2013) using the ‘import_biom’ function. Metadata was imported using the ‘import_qiime_sample_data’ and merged with the ASV table, representative sequences, and tree into a phyloseq object. Three samples with very low ASV counts (ASV counts = 1, 504, and 3102) were omitted from downstream analyses. Of the remaining samples (n = 71), the lowest ASV count in a sample was 14, 545.

Richness (observed ASVs), Shannon diversity index, and Faith’s phylogenetic distance (FPD) were calculated for all remaining samples (n = 71) with phyloseq and the ‘estimate_pd’ function from the btools package. ASV counts were then normalized using cumulative sum scaling (Paulson et al., 2013) and beta-diversity was analyzed using generalized UniFrac distances (Lozupone et al., 2011; Chen et al., 2012). From these distances, non-metric multidimensional scaling (NMDS) was performed and plotted, and permutational multivariate analysis of variance (PERMANOVA) was used to test for significant differences in community structure using the vegan (Oksanen et al., 2019) and pairwiseAdonis (Arbizu, 2017) packages. To ensure significant differences were not the result of unequal dispersions of variance between groups, permutational analysis of dispersion (PERMDISP) were conducted for all significant PERMANOVA outcomes using vegan. Additionally, hierarchal clustering was performed on generalized UniFrac distances using Ward’s agglomeration method (Murtagh and Legendre, 2014) and the ‘hclust’ function. Dendrograms were created from the hierarchal clustering results using the ‘ggdendro’ package. Further, the relative abundances of normalized ASVs within each sample were calculated and plotted using phyloseq.

To assess changes in microbial taxa with antifungal properties, ASVs were aligned using BLAST+ v2.11.0 to the comprehensive Antifungal Isolates Database of amphibian skin-associated bacteria that have been tested for antifungal properties (Woodhams et al., 2015). This database consists of 16S rRNA gene sequences from the bacterial isolates tested for antifungal properties. ASVs were aligned using the ‘blastn’ function, a percent identify cutoff of 99%, and a minimum e-value of 10^-6^. ASVs with positive alignments based on these thresholds were then identified and separated in phyloseq. The richness and diversity of antifungal ASVs were calculated and plotted as described above, and the relative abundance of antifungal ASVs was calculated from the CSS-normalized counts described above.

### Statistical analysis

Unless specified otherwise, R version 4.2.1 was used for statistical analysis of data. Pairwise Wilcoxon rank-sum tests were performed with a Benjamini-Hochberg correction for multiple comparisons. Differences in beta-diversity were tested using pairwise PERMANOVA with a Benjamini-Hochberg correction for multiple comparisons and 9,999 permutations. Additionally, pairwise PERMDISPs were carried out for all significant PERMANOVA outcomes using 9,999 permutations to test for differences in the variability of dispersions.

### Data availability

All sequence reads were made available through BioProject PRJNA1013348 at the NCBI’s Sequence Read Archive. The code and instructions for the bioinformatic and statistical analyses can be found at this GitHub repository: https://github.com/ljpinnell/SpringPeeper_SkinMicrobiome.

## Results

### Sequencing metrics

Samples included in our analysis (n = 71) had a range of 14,545 ASVs to 126,957 ASVs per sample and an average of 60,391 ASVs per sample. While only ~10% of all ASVs were classified at the level of genus, greater than 99% of all ASVs were classified at the ranks of family, order, class, and phylum across all samples (**Supplementary Table S1**).

### Shifts in overall microbial community diversity and composition

**Figure 1:**
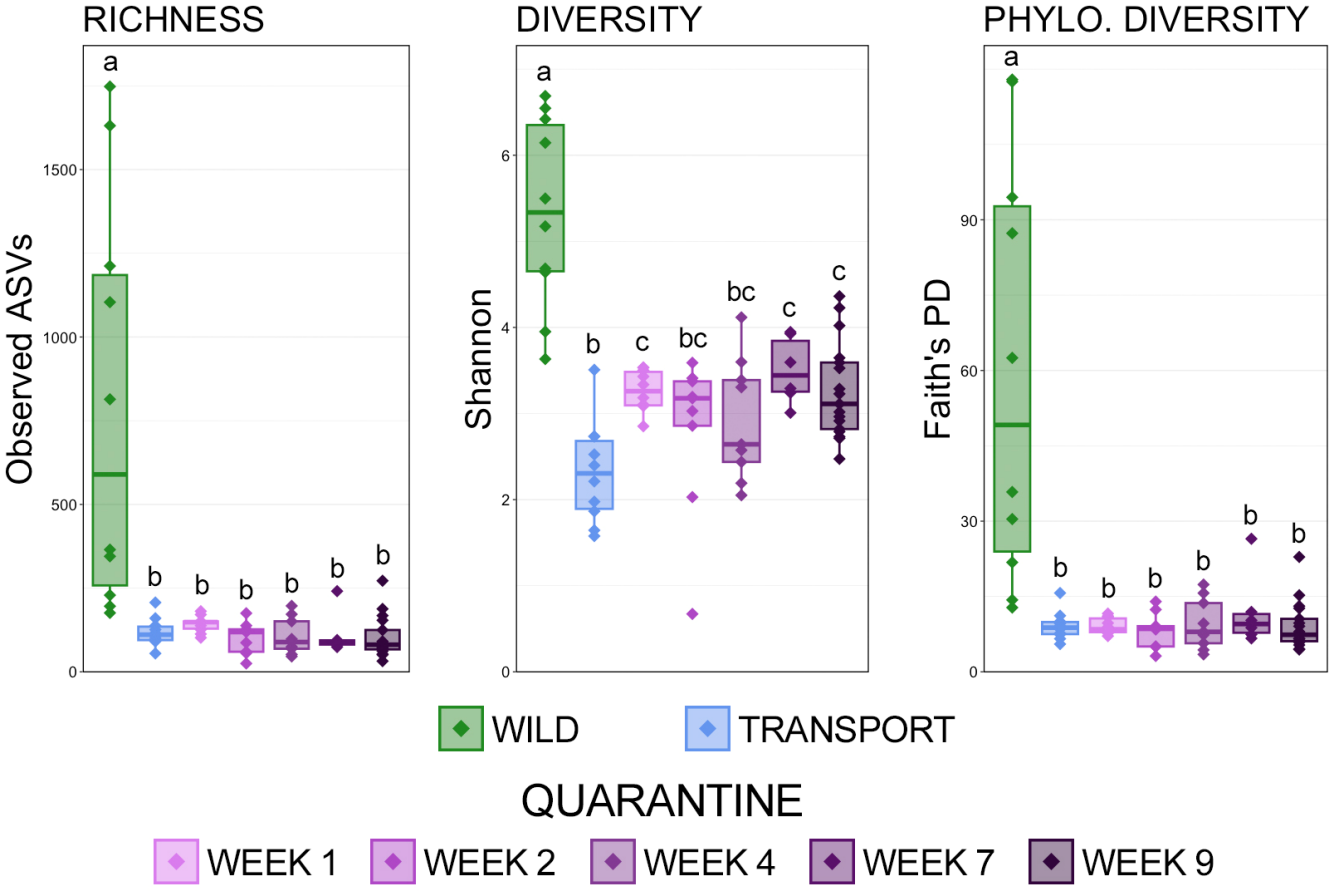
Boxplots demonstrating the richness (observed ASVs), diversity (Shannon), and phylogenetic diversity (Faith’s phylogenetic distance) between skin microbial communities from each of the 7 sampling timepoints. Significant differences in alpha-diversity are illustrated by different letters (pairwise Wilcoxon rank-sum ANOVA with Benjamini-Hochberg correction, n = 6-17, p < 0.05).

The comparison of observed ASVs, Shannon’s index, and Faith’s phylogenetic distance showed that richness, diversity, and phylogenetic diversity of skin microbial communities decreased significantly immediately after moving from the wild to a transport tube and remained significantly lower throughout a frog’s time in quarantine (**Figure 1**; pairwise Wilcoxon rank-sum ANOVA with Benjamini-Hochberg correction, n = 6-17, p < 0.05). While there were no differences between richness and phylogenetic diversity between transport and the end of the 9-week quarantine period, diversity did rebound and increase over a frog’s time in quarantine, albeit nowhere near the original diversity in the wild (**Figure 1**; pairwise Wilcoxon rank-sum ANOVA, n= 6-17, p < 0.05).

**Figure 2:**
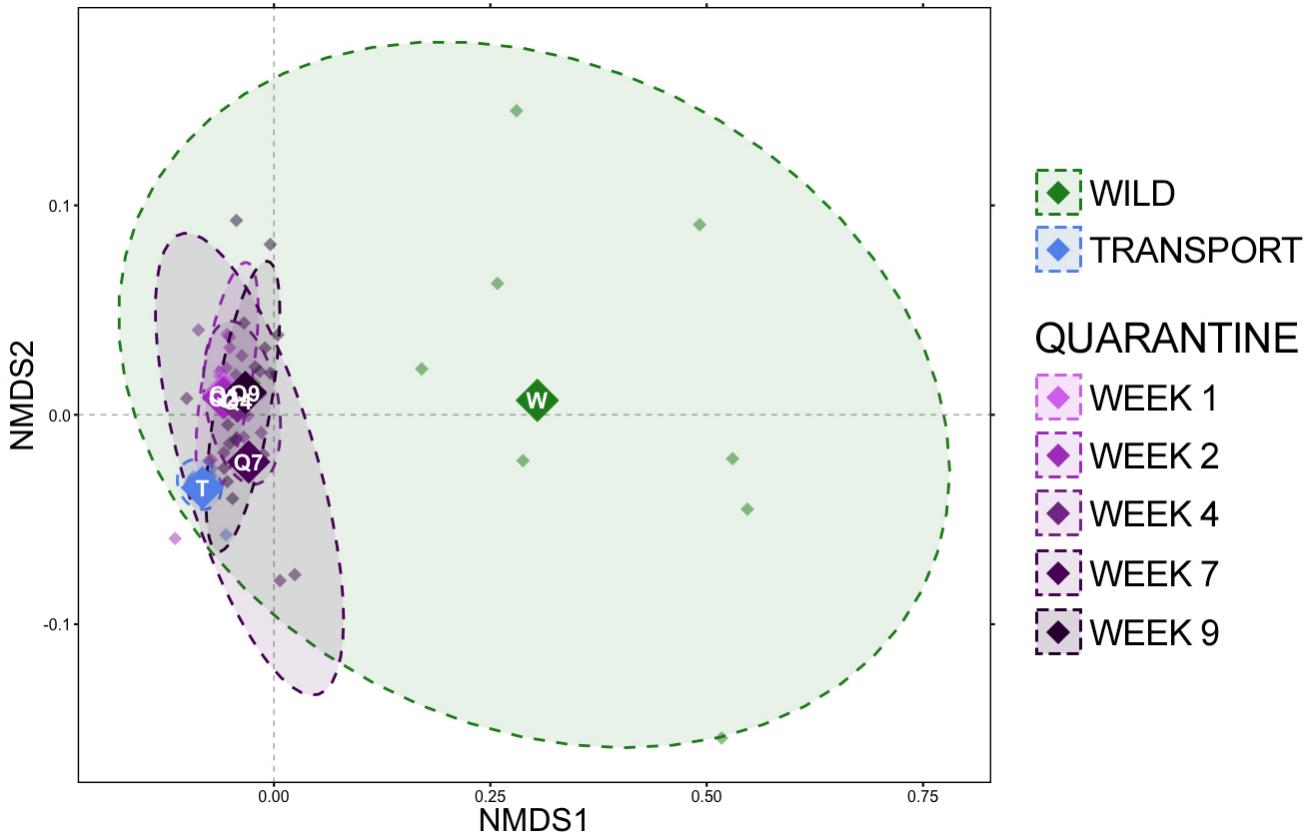
Non-metric multidimensional scaling (NMDS) of generalized UniFrac distances illustrating differences in overall microbial community structure between sampling timepoints. The NMDS demonstrates clustering of 16S rRNA gene sequences from frog skin communities sampled in the wild (green) after transport (blue) or during a 9-week quarantine period (5 shades of purple). The large opaque points represent the centroid for communities from each timepoint, while the smaller and more transparent points represent the individual frogs at each timepoint. Dashed lines and shaded areas represent 90% confidence intervals.

Based on generalized UniFrac distances, a very distinct shift in community composition occurred upon moving from the wild to a transport tube (**Figure 2**; **Supplementary Table S2**; pairwise PERMANOVA with Benjamini-Hochberg correction, R^2^ = 0.421, p = 0.001). Community composition remained very different during quarantine from that of frogs in the wild (**Figure 2**; **Supplementary Table S2**), but towards the end of the quarantine period (week 7 and week 9), communities were slightly more similar to wild communities than during transport and the first week in quarantine as demonstrated by lower percent variation explained (**Figure 2**; **Supplementary Table S2**; e.g., wild v. QW1 R^2^ = 0.423, wild v. QW9 R^2^ = 0.303). Community composition during transport was significantly different from all quarantine timepoints (**Figure 2**; **Supplementary Table S2**; pairwise PERMANOVA with Benjamini-Hochberg correction, p = 0.001). Community composition shifted while in quarantine, with the largest differences in community composition occurring between the first week in quarantine versus later weeks (i.e., QW1 v QW4 R^2^ = 0.315). After week 4, changes in composition were either subtle and/or not significant (**Figure 2**; **Supplementary Table S2**; QW4 v. QW7 R^2^ = 0.114, p = 0.026; QW4 v. QW9 R^2 =^ 0.062, p = 0.077) and there was no difference in community composition between week 7 and week 9 of the quarantine period (**Figure 2**; **Supplementary Table S2**; pairwise PERMANOVA with Benjamini-Hochberg correction, R^2^ = 0.050, p = 0.328).

**Figure 3:**
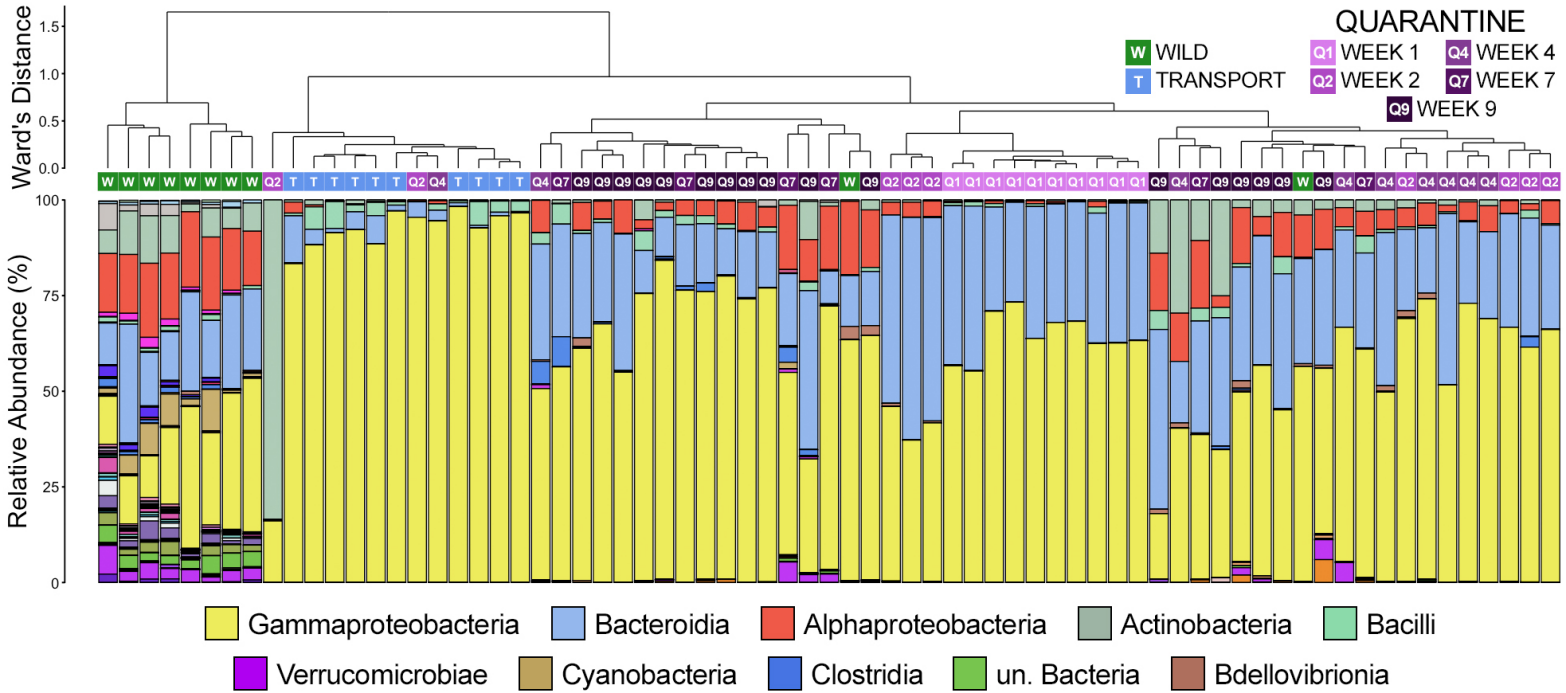
Dendrogram displaying the relatedness of skin microbial communities from different timepoints based on normalized ASVs. Hierarchal clustering was performed on generalized UniFrac distances using Ward’s agglomeration method. Green boxes represent communities from animals sampled in the wild, blue boxes represent those sampled after transport, and purple boxes represent communities from frogs in quarantine. The bar plot illustrates the relative abundance of microbial classes with each individual sample. The 10 most abundant classes are displayed in the legend.

Hierarchal clustering revealed that skin communities sampled in the wild were the most different from any others and, with two exceptions, formed their own unique clade (**Figure 3**). Communities sampled after transport were the next most unique communities, which were all found within one sub-clade made up almost exclusively of samples collected after transport (12/14 clade members; **Figure 3**). Communities sampled after 1 week of quarantine also formed their own clade, while communities after two weeks were largely interspersed among other clades made up of quarantine or transport samples. The main driver of clade formation appears to be the relative abundance of members of the class Gammaproteobacteria, which increased substantially after moving from the wild (**Figure 3**). Gammaproteobacteria was particularly predominant within communities collected after transport, while members of Alphaproteobacteria and Bacteroidia went from being among the top three classes in wild communities to virtually absent (**Figure 3**). During quarantine, members of Gammaproteobacteria were less abundant than after transport but still substantially more abundant as compared to wild communities (**Figure 3**). Members of Bacteroidia rebounded after the first week of quarantine to levels higher than in the wild, while Alphaproteobacteria returned to similar abundances to the wild later during the quarantine period (**Figure 3**).

### Changes in abundant taxa after moving to a managed environment

**Figure 4:**
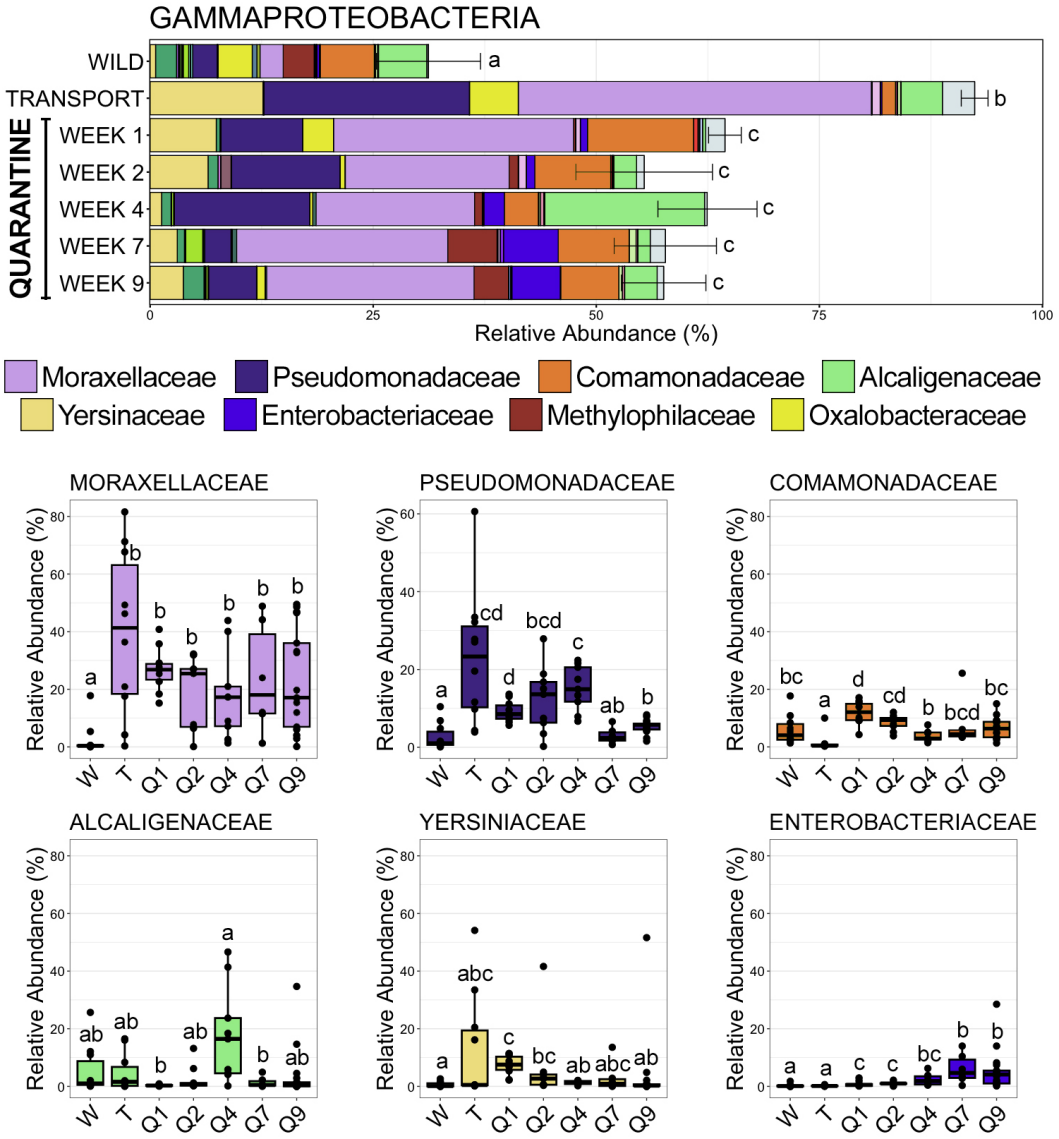
Bar plots demonstrating the mean relative abundance of all families within the class Gammaproteobacteria at each of the seven timepoints. Error bars represent standard error of the mean for the class Gammaproteobacteria. Boxplots demonstrating the relative abundance of the six most abudant Gammaproteobacteria families. For both plots, significant differences between timepoints are illustrated by different letters (pairwise Wilcoxon rank-sum, n = 6-17, p < 0.05).

**Figure 5:**
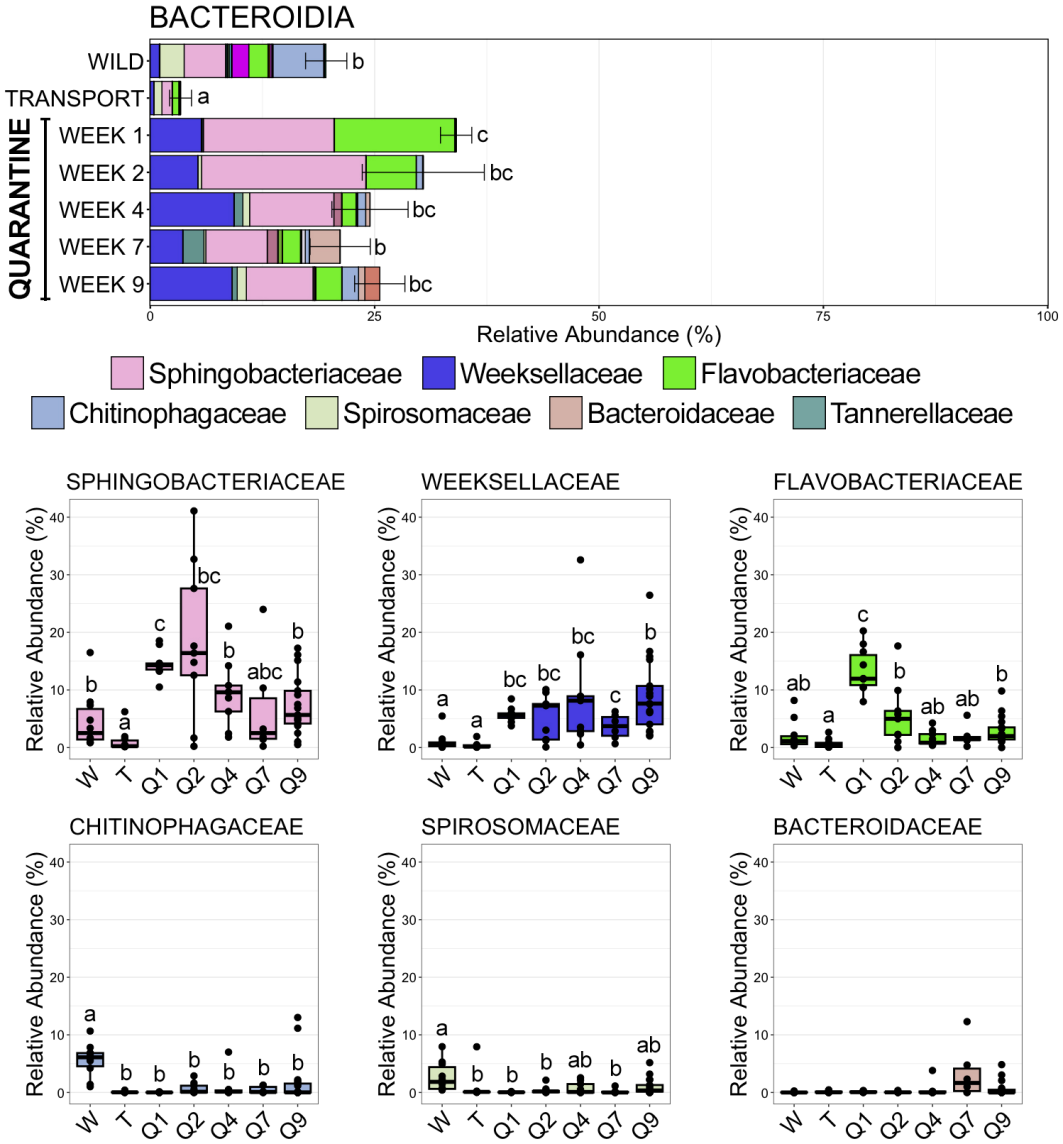
Bar plots demonstrating the mean relative abundance of all families within the class Bacteroidia at each of the seven timepoints. Error bars represent standard error of the mean for the class Bacteroidia. Boxplots demonstrating the relative abundance of the six most abudant Bacteroidia families. For both plots, significant differences between timepoints are illustrated by different letters (pairwise Wilcoxon rank-sum, n = 6-17, p < 0.05).

Moving from the wild to a managed environment resulted in drastic changes to members of the classes Gammaproteobacteria and Bacteroidia. Gammaproteobacteria, the most abundant class across all samples, became overwhelmingly dominant after frogs spent time in a transport tube (92.40% RA ± 1.50 SEM; SI Data) as compared to the wild (31.20% RA ± 5.82 SEM; SI Data), before stabilizing while in quarantine (QW1 64.42% RA ± 1.83 SEM; QW2 55.36% RA ± 7.66 SEM; QW4 62.46% RA ± 5.55 SEM; QW7 57.74% RA ± 5.73 SEM; QW9 57.55% RA ± 4.88 SEM; SI Data) at relative abundances between those in the wild and transport tube (**Figure 4**; pairwise Wilcoxon rank-sum ANOVA with Benjamini-Hochberg correction, n = 6-17, p < 0.05). The spike in Gammaproteobacteria abundance after being in the transport tube was largely the result of significant increases in relative abundance of Moraxellaceae and Pseudomonadaceae, the two most abundant families within Gammaproteobacteria (**Figure 4**; pairwise Wilcoxon rank-sum ANOVA with Benjamini-Hochberg correction, n = 6-17, p < 0.05). For example, Moraxellaceae went from being detected in only two of ten wild communities to being the most abundant family (>40% RA) across any lineage after transport (**Figure 4**, **Figure 5**; SI Data). Conversely, the third most abundant Gammaproteobacteria family, Comamonadaceae, decreased following the move to a transport tube (**Figure 4**; pairwise Wilcoxon rank-sum ANOVA with Benjamini-Hochberg correction, n = 6-17, p < 0.05). Enterobacteriaceae increased over the time course of the study, reaching significantly higher relative abundance during the seventh and ninth week of quarantine as compared to within earlier (i.e., wild, transport) communities (**Figure 4**; SI Data; pairwise Wilcoxon rank-sum ANOVA with Benjamini-Hochberg correction, n = 6-17, p < 0.05).

Bacteroidia, the second most abundant class across all samples, demonstrated the opposite trend to Gammaproteobacteria; significantly decreasing in relative abundance after moving from the wild to the transport tube (**Figure 5**; SI Data; pairwise Wilcoxon rank-sum ANOVA with Benjamini-Hochberg correction, n = 6-17, p < 0.05). Bacteroidia was the second most abundant class within skin communities in the wild (19.60% RA ± 2.30 SEM; SI Data), but after transport members of Bacteroidia comprised less than 5% of skin communities (3.38% RA ± 1.22 SEM; SI Data). Interestingly, Bacteroidia rebounded during the 9-week quarantine (QW1 34.08% RA ± 1.74 SEM; QW2 30.42% RA ± 6.80 SEM; QW4 24.48% RA ± 4.25 SEM; QW7 21.16% RA ± 3.35 SEM; QW9 25.57% RA ± 2.80 SEM; SI Data) and became more abundant than it was in skin communities in the wild (**Figure 5**; pairwise Wilcoxon rank-sum ANOVA with Benjamini-Hochberg correction, n = 6-17, p < 0.05). Decreases in the relative abundances of three Bacteroidia families were the main reason for its drop after transport. Chitinophagaceae comprised over 5% of the overall community in the wild, but significantly decreased and was rarely even detected in communities after transport or during any point of quarantine (**Figure 5**; SI Data; pairwise Wilcoxon rank-sum ANOVA with Benjamini-Hochberg correction, n = 6-17, p < 0.05). Sphingobacteriaceae, the most abundant Bacteroidia family, was in significantly lower relative abundance following transport as compared to in the wild, but then rebounded to abundances similar to or higher than those in the wild while in quarantine (**Figure 5**; pairwise Wilcoxon rank-sum ANOVA with Benjamini-Hochberg correction, n = 6-17, p < 0.05). Spirosomaceae also decreased significantly in relative abundance after moving to the transport tube and rebounded slightly to abundances between those in the wild and transport after the 9-week quarantine period (**Figure 5**; pairwise Wilcoxon rank-sum ANOVA with Benjamini-Hochberg correction, n = 6-17, p < 0.05). Weeksellaceae, the second most abundant family within Bacteroidia, was only sparsely detected within skin communities in the wild or after transport but increased in abundance during the quarantine period to comprise nearly 10% of the overall skin community at the end of the 9-week quarantine (**Figure 5**; pairwise Wilcoxon rank-sum ANOVA with Benjamini-Hochberg correction, n = 6-17, p < 0.05).

### Increased relative abundance but decreased diversity of antifungal taxa in a managed environment

**Figure 6:**
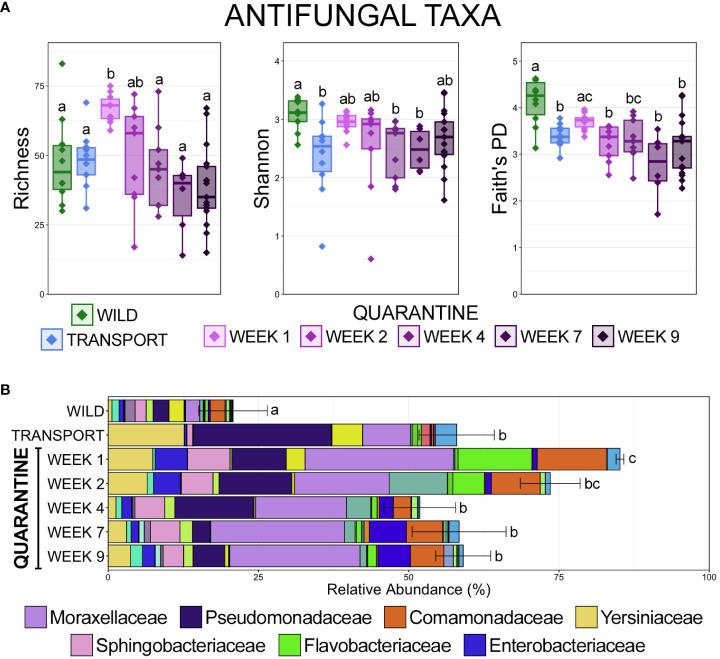
**(A)** Boxplots demonstrating the richness (observed ASVs), diversity (Shannon), and phylogenetic diversity (Faith’s phylogenetic distance) of microbial taxa with known antifungal properties from skin communities at each of the 7 sampling timepoints. **(B)** Bar plots demonstrating the relative abundance of microbial taxa with known antifungal properties from skin communities at each of the 7 sampling timepoints. Error bars represent the standard error of the mean for the cumulative total relative abundance of all antifungal taxa at each timepoint. Significant differences are illustrated by different letters (pairwise Wilcoxon rank-sum ANOVA with Benjamini-Hochberg correction, n = 10, p < 0.05).

Alignment to a database comprised of 16S rRNA gene sequences from bacteria with known antifungal properties revealed that the richness of antifungal taxa increased significantly after transport from the wild, but stabilized back to a similar richness within communities in the wild (**Figure 6A**; pairwise Wilcoxon rank-sum ANOVA with Benjamini-Hochberg correction, n = 6-17, p < 0.05). Despite an increased richness after transport compared to in the wild, the diversity and phylogenetic diversity of antifungal taxa was significantly lower after transport, and phylogenetic diversity remained significantly lower after the 9-week quarantine period (**Figure 6A**; pairwise Wilcoxon rank-sum ANOVA with Benjamini-Hochberg correction, n = 6-17, p < 0.05). The higher richness but lower diversity after transport as compared to in the wild is the result of lower evenness of anti-fungal taxa within communities in managed environments, with anti-fungal taxa being largely contained to Pseudomonaceae, Yersiniaceae, and Moraxellaceae after transport (**Figure 6B**). Despite being less diverse, antifungal taxa represented significantly more of the overall microbial community after transport as compared to communities in the wild (**Figure 6B**; pairwise Wilcoxon rank-sum ANOVA with Benjamini-Hochberg correction, n = 6-17, p < 0.05). The relative abundance of antifungal taxa increased significantly early in the quarantine period, before returning to abundances similar to after transport but higher than those in the wild (**Figure 6B**; pairwise Wilcoxon rank-sum ANOVA with Benjamini-Hochberg correction, n = 6-17, p < 0.05). Antifungal taxa within Moraxellaceae were the most abundant throughout the quarantine period, while antifungal taxa from Pseudomonadaceae, which were the most predominant immediately after transport, decreased considerably during quarantine. Interestingly, antifungal taxa from Flavobacteriaceae spiked during the first two weeks in quarantine before substantially decreasing (**Figure 6B**).

## Discussion

This unique study provides a detailed analysis of how the skin associated microbiome of spring peeper frogs changed as the animals move from the wild to being managed under human care at a large public display aquarium. While managed collections play important roles in education, research, and conservation efforts of amphibians it is critical we understand how collection and handling affects their skin microbiomes. Our findings demonstrate that changes in environment conditions during transport from the wild had drastic impacts on the diversity and composition of skin microbial communities in spring peeper frogs that lasted throughout a 9-week quarantine period. Importantly, microbial taxa with anti-fungal properties became less diverse but more predominant within skin microbial communities following transport from the wild. However, whether a less diverse set of anti-fungal microbes equates to a loss of resilience to fungal pathogens remains unknown and further work is needed to answer this important question.

After these frogs were moved from the wild into quarantine, the microbial community shifted quickly and drastically. Similarly, *Rana cascadae* tadpoles experienced microbiota differences depending on environment as well as life stage (Kueneman et al., 2014), suggesting that water plays a crucial role in amphibian skin microbiome as wetland sites in the *Rana* tadpoles explained significant variation. Future studies should evaluate the microbiome of water sources of frogs, in addition to the skin microbiome.

The underlying mechanism driving the community structure shifts cannot be determined from the data collected in this study, but it is striking that the largest shift happened immediately after removal from the wild. This may suggest that a host response like glucocorticoid release may play a role. In eastern newts (*Notophthalmus viridescens*), corticosterone was not considered to be a major factor in immunity (Pereira et al., 2023), but in salamanders (*Plethodon shermani*) differences were seen between corticosterone and chytridiomycosis resistance (Fonner et al., 2017), suggesting that this relationship may need to be further evaluation, specifically in frogs. Additionally, longitudinal sampling of the same individuals throughout the transportation process would allow for better temporal resolution, identifying shifts in composition and taxa, and improved statistical power.

Over the quarantine period, microbial diversity on the skin of the frogs decreased, with the largest difference occurring within the first week. Managed habitats typically have lower environmental microbial diversity than natural environments and given the susceptibility of amphibian skin to changing environmental conditions (Houlahan et al., 2000; Varga et al., 2019) it follows that microbial diversity would decrease following a move to a managed habitat. Previous research on fire-bellied toads (*Bombina orientalis*) has demonstrated that wild toads had more diverse skin microbial communities than captive toads (Bataille et al., 2016). Further, decreases in diversity are commonly observed among host-associated microbial communities living in managed environments when compared to wild environments across multiple species (Pinnell et al., 2020; Dallas and Warne, 2023).

Additionally, once frogs were in quarantine, they received topical treatment with a standard antiparasitic, ivermectin. Ivermectin, a macrocytic lactone, results in parasitic paralysis and death secondary to ion channel binding (Johnson-Arbor, 2021). Alternative anthelminthic topical treatments, such as levamisole, have been efficacious in the reduction of nematodes in toads (Bianchi et al., 2014). Future studies should compare skin microbiome between different topical antiparasitic medications as well as a control group.

The most predominant bacterial classes identified across all skin communities were Gammaproteobacteria, Alphaproteobacteria, and Bacteroidia, which has previously been described in other amphibian species (Kueneman et al., 2014). Gammaproteobacteria increased in abundance after transport, largely as a result of a dramatic increase in its most abundant family Moraxellaceae. Interestingly, Moraxellaceae was the dominant family seen on farmed Chinese tiger frogs (*Hoplobatrachus rugulosus*) with ulcerated skin (Hu et al., 2022). No histopathology samples were collected from the frogs in the current study to quantify skin health, however, clinically, all frogs continued to thrive after completion of this study. In contrast to Gammaproteobacteria, Bacteroidia decreased significantly after transport from the wild. Previous work by our group demonstrated a similar decrease in Bacteroidia in yellow stingray-associated microbial communities after moving to managed care (Pinnell et al., 2020). Here, however, Bacteroidia appeared to rebound very quickly once frogs moved into quarantine; back to their relative abundance in the wild by the first week or quarantine. Conversely, members of other classes – Actinobacteria and Alphaproteobacteria for example – didn’t start rebounding until at least the second week of quarantine. Because of its seemingly important role very early in the quarantine period, future research is needed to fully evaluate the role of Bacteroidia during different environmental conditions, such as humidity, substrate, and dietary change associated with transport from the wild to managed care.

After moving from the wild to a managed habitat, the diversity of microbes with anti-fungal properties decreased. Yet, the proportion of the overall skin community these microbes comprised increased dramatically. Typically, lower diversity is equated with lower resistance (insensitivity to disturbance) and less resilience (rate of recovery after disturbance) in a microbial community (Shade et al., 2012). However, whether the lower microbial diversity observed here was reflected in the anti-fungal gene pool is impossible to ascertain using 16S rRNA gene sequencing. Given the importance of anti-fungal metabolites produced by microbial populations on amphibian skin in providing protecting against fungal infections like *Bd* (Rebollar et al., 2020; Woodhams et al., 2020), further studies targeting anti-fungal gene or protein expression in amphibian microbial communities following a move to managed care are warranted.

## Data availability statement

The datasets presented in this study can be found in online repositories. The names of the repository/repositories and accession number(s) can be found below: https://www.ncbi.nlm.nih.gov/, BioProject PRJNA1013348 https://github.com/ljpinnell/SpringPeeper_SkinMicrobiome, GitHub.

## Ethics statement

The frogs in this study were collected under permit from the Illinois Department of Natural Resources (Permit Number: A15.1006). The study was conducted in accordance with the local legislation and institutional requirements.

## Author contributions

LK: Writing – review & editing, Writing – original draft, Investigation. WV: Writing – review & editing, Supervision, Project administration, Funding acquisition, Conceptualization. FO: Writing – review & editing, Validation, Supervision, Project administration, Methodology, Investigation. CE: Writing – review & editing, Validation, Data curation. MS: Writing – review & editing, Methodology, Investigation, Conceptualization. LP: Writing – review & editing, Writing – original draft, Visualization, Supervision, Project administration, Methodology, Investigation, Funding acquisition, Formal analysis, Data curation.
