# Supplementary material for: Transport from the wild rapidly alters the diversity and composition of skin microbial communities and antifungal taxa in spring peeper frogs
Source: Front Microbiomes. 2024 Apr 19;3:1368538. doi: 10.3389/frmbi.2024.1368538 (PMC12993632; doi:10.3389/frmbi.2024.1368538)
Supplement: Supplementary file 1 [file Table_1.docx]

**Table S1.** Proportion of ASVs classified at the taxonomic ranks of phylum, class, order, family, and genus for skin communities across all samples.

| **Phylum** | **Class** | **Order** | **Family** | **Genus** |
| --- | --- | --- | --- | --- |
| 99.51% | 99.48% | 99.42% | 99.23% | 9.56% |
