# Supplementary material for: Transport from the wild rapidly alters the diversity and composition of skin microbial communities and antifungal taxa in spring peeper frogs
Source: Front Microbiomes. 2024 Apr 19;3:1368538. doi: 10.3389/frmbi.2024.1368538 (PMC12993632; doi:10.3389/frmbi.2024.1368538)
Supplement: Supplementary file 2 [file Table_2.docx]

**Table S2**. PERMANOVA and PERMDISP results from comparisons between timepoints based on generalized UniFrac values. Significant values are bolded (P < 0.05).

| **Test** | **Df** | **SS** | **Pseudo-F** | **R^2^** | **p-adj.** | **PERMDISP (p-adj.)** |
| --- | --- | --- | --- | --- | --- | --- |
| **WILD** |  |  |  |  |  |  |
| Wild v. Transport | 1 | 0.896 | 13.081 | 0.421 | **0.001** | **0.0001** |
| Wild v. QW1 | 1 | 0.823 | 13.177 | 0.423 | **0.001** | **0.0001** |
| Wild v. QW2 | 1 | 0.666 | 8.306 | 0.328 | **0.001** | **0.0001** |
| Wild v. QW4 | 1 | 0.620 | 7.868 | 0.316 | **0.001** | **0.0001** |
| Wild v. QW7 | 1 | 0.432 | 4.394 | 0.239 | **0.010** | **0.0019** |
| Wild v. QW9 | 1 | 0.753 | 10.847 | 0.303 | **0.001** | **0.0001** |
| **TRANSPORT** |  |  |  |  |  |  |
| Transport v. QW1 | 1 | 0.287 | 25.785 | 0.589 | **0.001** | **0.0047** |
| Transport v. QW2 | 1 | 0.233 | 8.977 | 0.346 | **0.001** | 0.0816 |
| Transport v. QW4 | 1 | 0.232 | 9.437 | 0.357 | **0.001** | **0.0426** |
| Transport v. QW7 | 1 | 0.205 | 6.354 | 0.312 | **0.001** | **0.0035** |
| Transport v. QW9 | 1 | 0.349 | 10.756 | 0.301 | **0.001** | **0.0023** |
| **QUARANTINE** |  |  |  |  |  |  |
| QW1 v. QW2 | 1 | 0.067 | 3.452 | 0.169 | **0.001** | **0.0007** |
| QW1 v. QW4 | 1 | 0.141 | 7.825 | 0.315 | **0.001** | **0.0001** |
| QW1 v. QW7 | 1 | 0.170 | 6.972 | 0.332 | **0.001** | **0.0001** |
| QW1 v. QW9 | 1 | 0.188 | 6.707 | 0.212 | **0.001** | **0.0001** |
| QW2 v. QW4 | 1 | 0.060 | 1.744 | 0.099 | **0.049** | 0.9354 |
| QW2 v. QW7 | 1 | 0.107 | 2.396 | 0.156 | **0.008** | 0.1937 |
| QW2 v. QW9 | 1 | 0.099 | 2.530 | 0.095 | **0.002** | 0.3887 |
| QW4 v. QW7 | 1 | 0.072 | 1.667 | 0.114 | **0.026** | 0.0911 |
| QW4 v. QW9 | 1 | 0.061 | 1.589 | 0.062 | 0.077 | 0.2808 |
| QW7 v. QW9 | 1 | 0.050 | 1.104 | 0.050 | 0.328 | 0.3456 |

Abbreviations: v., versus; QW, quarantine week; Df, degrees of freedom
